# Aromatic components and endophytic fungi during the formation of agarwood in *Aquilaria sinensis* were induced by exogenous substances

**DOI:** 10.3389/fmicb.2024.1446583

**Published:** 2024-08-21

**Authors:** Shengjiang Pang, Weiwei Zhao, Qingqing Zhang, Zuwei Tian, Dan Wu, Shuokun Deng, Pei Zhang, Zhongguo Li, Shiling Liu, Baoguo Yang, Guihua Huang, Zaizhi Zhou

**Affiliations:** ^1^Experimental Center of Tropical Forestry, Chinese Academy of Forestry, Pingxiang, China; ^2^Research Institute of Tropical Forestry, Chinese Academy of Forestry, Guangzhou, China; ^3^College of Forestry, Nanjing Forestry University, Nanjing, China; ^4^Fujian Academy of Forestry, Fuzhou, China; ^5^Guangxi International Zhuang Medical Hospital, Guangxi University of Chinese Medicine, Nanning, China

**Keywords:** *Aquilaria sinensis*, aromatic components, endophytic fungal diversity, agarwood-induction technique, induction time, agarwood

## Abstract

The process of formation of aromatic components for agarwood in *Aquilaria sinensis* is closely related to endophytic fungi and the result of complex multiple long-term joint interactions with them. However, the interactions between the aromatic components and endophytic fungi remain unclear during the formation of agarwood. In this study, precise mixed solution of hormones, inorganic salts, and fungi was used to induce its formation in *A. sinensis*, and sample blocks of wood were collected at different times after inoculation. This study showed that the aromatic compounds found in the three treatments of *A. sinensis* were primarily chromones (31.70–33.65%), terpenes (16.68–27.10%), alkanes (15.99–23.83%), and aromatics (3.13–5.07%). Chromones and terpenes were the primary components that characterized the aroma. The different sampling times had a more pronounced impact on the richness and diversity of endophytic fungal communities in the *A. sinensis* xylem than the induction treatments. The species annotation of the operational taxonomic units (OTUs) demonstrated that the endophytic fungi were primarily composed of 18 dominant families and 20 dominant genera. A linear regression analysis of the network topology properties with induction time showed that the interactions among the fungal species continued to strengthen, and the network structure tended to become more complex. The terpenes significantly negatively correlated with the Pielou evenness index (*p* < 0.05), while the chromones significantly positively correlated with the OTUs and Shannon indices.

## 1 Introduction

Agarwood, known as “diamond in the wood,” is primarily derived from a species of *Aquilaria* in the family Thymelaeaceae. Agarwood contains aromatic oils in its wood, and these types of *Aquilaria* trees primarily grow in Cambodia, China, Laos, Malaysia, Vietnam and Thailand, as well as in India and other southeastern Asian regions (Xu et al., 2016; Wang et al., 2018; Azren et al., 2019; Wang et al., 2019). Among these components, terpenoids, chromones and aromatics are the characteristic aromatic components of agarwood, which are related to its quality (Triadiati and Carolina, 2016). Additionally, the wood contains some fatty acids and alkanes. These aromatic components are not detectable in the fresh parts of healthy *Aquilaria* trees but might form in those that have been wounded or colonized by microorganisms. Furthermore, studies have demonstrated that fungi can effectively induce the formation of agarwood, and the type of compounds produced is closely related to the type of fungus used for the induction (Mei et al., 2013). The inoculation of *Rigidoporus vinctus*, *Phaeocremonium rubrigenum*, *Paraconiothyrium* var*iabile*, *Lasiodiplodia theobromae* or *Fusarium oxysporum* in the xylem of *Aquilaria sinensis* has been demonstrated to effectively induce the formation of agarwood. This phenomenon has been observed in the case of *A. sinensis* induced by *R. vinctus*. The resulting incense had a leachate content of 38.9%, with a relative content of terpenoids up to 22.76%. The essential oils of the agarwood, induced by *Fusarium acuminatum*, contained 76.77% terpenoids (Chen et al., 2018; Azren et al., 2019; Liu et al., 2022; Zhang P. et al., 2022; Zhang Z. et al., 2022). The treatment of *A. malaccensis* with *Penicillium polonicum* for 3 months resulted in the production of agarospirol (Chhipa and Aushik, 2017). The presence of aromatics, including benzylacetone and 4-(p-tolyl) butan-2-one, was observed in samples collected 8 months after inoculation with *Xylaria* sp. in the *A. sinensis* xylem. Benzylacetone and 6,7-dimethoxy-2-(2-phenylethyl) chromone were identified in incense produced by the inoculation of *Lasiodiplodia theobromae* and *Fusarium*. Furthermore, inoculation with *Fusarium solani* was also used successfully to obtain superior agarwood (Cui et al., 2013; Chen X. et al., 2017; Chen X. Y. et al., 2017; Faizal et al., 2017). However, the quality of the resulting agarwood remained variable, with considerable differences observed between the samples. This variation may be attributed to the diversity of endophytic fungi present in *Aquilaria* trees as previously described (Mohamed et al., 2014a,b; Ma et al., 2021; Liu et al., 2022, 2024).

Endophytic fungi are a group of microorganisms that reside within healthy plant tissues or organs at a specific stage of their life cycle. They do not cause overt disease symptoms in the host plant and perform functions that promote the growth and development of the plant, enhance its resistance, and facilitate the accumulation of secondary metabolites (Gómez and Luiz, 2018; Ye et al., 2020). It has been demonstrated that *Fusarium* is a dominant genus of the endophytes of *Euphorbia pekinensis* and *Dendrobium officinale* Kimura et Migo, and its symbiosis with host plants can promote seed germination and seedling growth (Dai et al., 2008; Sujit et al., 2018). A study of the intercropping of *Penicillium funiculosum* with leguminous plants also showed that this fungus significantly increased the stem length, leaf area, and biomass indices of the latter by producing indole-3-acetic acid (IAA), which was highly effective at promoting growth (Abdul et al., 2011; Bilal et al., 2019). This indicates that endophytic fungi can biosynthesize phytohormones. Examples include *Aspergillus niger* that secretes gibberellin (GA_3_) and *Trichoderma* that also synthesizes IAA, which can regulate plant growth. Moreover, the endophytic fungal–plant symbionts can increase the ability of their host plant to tolerate severe stress (Manzur et al., 2022). Indeed, some plants cannot survive without the presence of endophytic fungi because they cannot tolerate the biotic or abiotic stress of the extreme environment in which they live.

Although some of the resistance characteristics of host plants are associated with the presence of endophytic fungi, the mechanism of how endophytic fungi assist plants in adapting to extreme environments remains poorly understood. It has been proposed that the cells of host plants that contain endophytic fungi can more rapidly scavenge the accumulation of reactive oxygen species through the biosynthesis of proline. For example, endophytic fungi of the genus *Epichloë* induce the synthesis of antioxidants, such as proline, that help *Hordeum brevisubulatum* tolerate flooding (Wang and Komatsu, 2018). Furthermore, endophytic fungi can mitigate the damage of stress on host plants by activating their antioxidant system, modulating the secretion of phytohormones or other bioactive substances, enhancing photosynthesis, and maintaining ion homeostasis (Clay and Holah, 1999; Meena et al., 2017; Ghorbani et al., 2018; Xiaoning et al., 2022). For example, Raid et al. (2022) demonstrated that maize (*Zea mays*) that had been colonized with *Stemphylium lycopersici* (Enjoji) Yamamoto had higher levels of IAA and an altered accumulation of secondary metabolites, including phenolics and flavonoids, which thereby improved the salt tolerance of this plant. The colonization of banana trees with *Piriformospora indica* has been shown to increase their tolerance to cold as shown by a decrease in the contents of malondialdehyde and hydrogen peroxide and an increase in the activities of superoxide dismutase and catalase (Li et al., 2021). Additionally, the contents of soluble sugars and proline increased. Membrane fluidity and the contents of essential amino acids, protein structures, secondary metabolites and osmolytes have also been found to be severely altered during the growth of plants in extreme environments (Zinn et al., 2010; Khan et al., 2013). Forest tree-fungal symbiotic relationships under stress have long been the subject of intensive research. However, there have been significantly fewer studies on endophytic fungi than on tufted mycorrhizal and ectomycorrhizal fungi. Furthermore, the potential of endophytic fungi to enhance the resilience of forest trees has not been fully explored, and there have been few studies on the effects of endophytic fungi on the physiology and accumulation of secondary metabolites on forest trees subjected to stress (Wu et al., 2017; Zhang et al., 2017). Therefore, it is imperative to conduct a comprehensive study on the ecological phenomenon of the symbiosis between endophytic fungi and plants under stress, along with the underlying mechanisms that facilitate this interaction.

The endophytic fungi of *Aquilaria* plants are primarily distributed in their xylem and are highly diverse. A total of 56 genera of endophytic fungi have been isolated and identified from species of *Aquilaria*; the most prevalent include species of *Fusarium*, *Lasiodiplodia*, *Colletotrichum*, and Trichoderma (Jayachandran et al., 2014; Turjaman et al., 2016). More endophytic fungi have been isolated from the fragrant parts of *Aquilaria* than from parts of the wood that do not form aromatics (Premalatha and Kalra, 2013; Xuyu et al., 2018). In addition, more and different types of endophytic fungi have been isolated from the fragrant parts of agarwood (*A. malaccensis*) than from the leaves and roots (Wang et al., 2009; Chen et al., 2018). The abundance and quality of endophytic fungi also vary among the fragrant parts of the same tree at different heights (Liu et al., 2019). Mohamed et al. (2014a,b) used real-time fluorescence quantitative PCR to show that the prevalence of endophytic at the wound sites of *A. sinensis* increased during the early stages of the formation of aromatics in the *A. sinensis* xylem, while the number of fungi in the already fragrant sites decreased during the later stages of the formation of these compounds. Based on this hypothesis, it can be inferred that a large number of fungi colonize the plant and accelerate the formation of aromatics during damage. Agarwood that is produced during the later stages of the formation of aromatics has antibacterial properties, and there is a decrease in the number of dominant fungal species. The process of the formation of aromatics in agarwood is closely related to endophytic fungi and may be the result of the joint action of multiple fungi.

Our previous research has shown that the technique used to induce the production of secondary compounds in agarwood, including solutions of hormones, inorganic salts, and fungi, can induce the formation of high-quality agarwood in the branches several meters away from an infused trunk (Zhao et al., 2024a,b). However, the diversity of aromatic components, endophytic fungi, and their interaction during the formation of agarwood in *A. sinensis* remains unclear. While the natural fungi and those artificially inoculated in agarwood may not have comparable effects (Monggoot et al., 2018; Mega et al., 2023), certain endophytic fungal groups isolated from *Aquilaria* trees may impact the aromatic quality of agarwood. Thus, it is necessary to compare the quality and diversity of endophytic fungi in the agarwood samples induced by exogenous substances to identify the active fungal group that can induce the volatile aromatic components of agarwood. Furthermore, this comparison may reveal if the grade of artificially inoculated agarwood can match that of naturally infected agarwood in terms of the fungi used.

In this study, a 10-year-old plantation of *A. sinensis* was selected as the subject, and the trees were induced by treating their trunks with different concentrations of a plant growth regulator (Ethephon), inorganic NaCl and calcium chloride (CaCl_2_), and a fungal solution. This study aimed to use high-throughput sequencing to identify the endophytic fungal groups. The next step focused on analyzing the diversity of fungi and the links between aromatic components and endophytic fungi. In this study, changes were observed in the groups of aromatic components and endophytic fungi found in the *A. sinensis* xylem. These changes help to identify the groups of endophytic fungi during the formation of aromatics and clarify the changing patterns of agarwood aromas.

## 2 Materials and methods

### 2.1 Sample source

The study was conducted at the Anshan Village, Shatang Town, Yulin City, Guangxi Zhuang Autonomous Regin, China (22°53′31″ N, 109°54′17.4″ E), with a subtropical monsoon climate. The average annual temperature in the region is 22.0°C, and there are 1,795 h of sunshine and 1,650 mm of precipitation a year on average. The climate is mild with synchronicity in the abundant precipitation and heat.

*Aquilaria sinensis* (Lour.) Spreng [Appraiser: Yingjian Li (College of Forestry, Guangxi University, Nanning 530,004, China), Appraisal Date: December 20, 2022] was the seed source of Hainan Province, China. A 10-year-old plantation of *A. sinensis* was selected for the agarwood induction experiment, and the trees selected were healthy and free of pests and diseases. The average diameter at breast height and tree height were 14.52 cm and 8.64 m, respectively. This study was conducted on November 25, 2022, and the sampling period from December 2022 to August 2023 was characterized by abundant precipitation and adequate hours of sunshine with typical seasonal characteristics.

### 2.2 Experimental design and sampling

This study utilized a completely random design with three different treatments, including Ethephon (a growth regulatory compound), NaCl, CaCl_2_, and a mixed solution of fungi that was prepared by mixing solutions of two fungal types in different volumes. Distilled water was used as the control. The composition of the liquid mixture for each treatment is shown in Table 1.

**Table 1:** Plant growth regulators, inorganic salts and fungi mixed solution treatments for induction.

Treatment	Ethephon (%)	NaCl (%)	CaCl_2_ (%)	Fungal solution
T1	2.0	2.0	1.5	*Melanotus flavolivens*:*Rigidoporus vinctus* 1:1 v/v
T2	2.0	1.0	1.0	*Melanotus flavolivens*:*Fusarium solani* 1:1 v/v
T3	2.0	1.5	0.5	*Rigidoporus vinctus*:*Fusarium solani* 1:1 v/v

Four directional infusion holes were drilled into the trunks of *A. sinensis* at 60–80 cm above the ground with a 5 mm drill bit on a sunny day. The infusion method was utilized to inject the induction solution into the trunks. Two 500 mL bags of mixed solution were suspended from each tree. A total of 30 trees were randomly selected for each treatment with three replicates.

**Figure 1:**
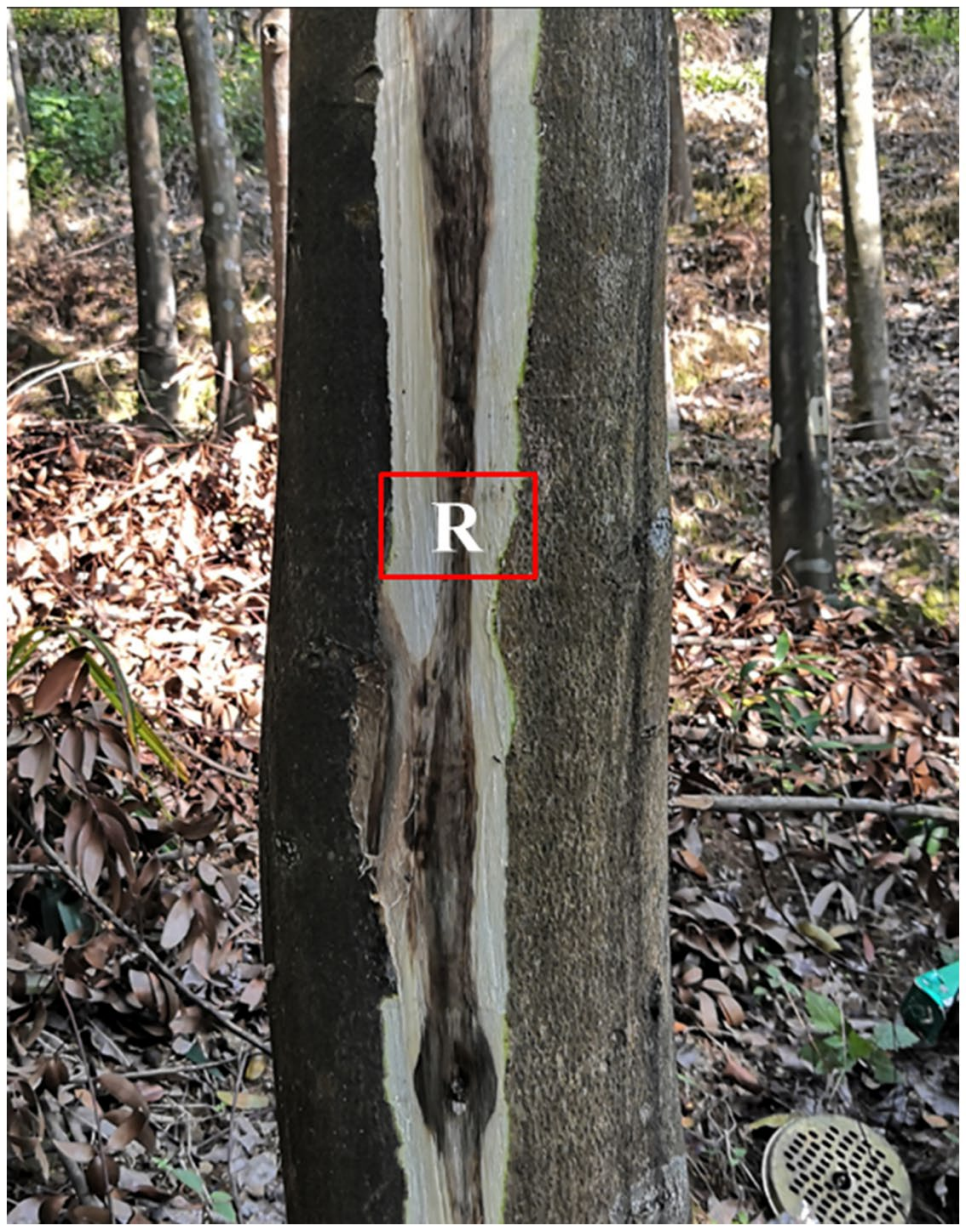
A wood sample collected from a wounded stem of *Aquilaria sinensis*. The sample was further sectioned based on the characteristics of its color and used to analyze the internal transcribed spacer. R, dark brown wood indicates the presence of agarwood.

The wood samples were collected on months 0, 3, 6, and 9 after induction. Three plants were randomly chosen from each treatment, and the wood samples were collected using an axe or chisel 30 cm above the infusion hole in the *A. sinensis* trunk (Figure 1). These samples were chipped into 1 × 2 cm pieces. Each was wrapped in aluminum foil and placed in liquid nitrogen until completely frozen. The samples were transported back to the laboratory and eventually stored at −80°C for subsequent measurements.

### 2.3 Determination of the aromatic components

Three *A. sinensis* trees were randomly selected for each induction treatment at 9 months. The dark wood that contained the aromatic oils was sampled using an axe or chisel and placed in liquid nitrogen until completely frozen. The samples were transported back to the laboratory and air-dried in a dark room. The samples of wood were shredded with a knife and ground into powder with a small desktop medicinal material grinder (YF-111B, Yongli Pharmaceutical Machinery Co., Ltd., Ruian city, China) to detect the composition of agarwood that had passed through a 40-mesh sieve.

A total of 2.0 g of samples were weighed and placed in a 50 mL centrifuge tube. A volume of 20 mL of 95% ethanol was added. The sample solution was treated by ultrasonication (Elmasonic P300H, Elma Ultrasonic, Wetzikon, Switzerland) at 35 kHz and 60°C in a water bath for 2 h. The suspension was obtained by passage through a 0.45 μm filter membrane, and the volumetric mixed solution was brought to 50 mL (Li et al., 2023). A volume of 10 mL of the sample was measured to determine its components using a Temperamental Co-Use Instrument (Agilent 7890B-5977A GC–MS; Agilent Technologies, Santa Clara, CA, United States) and column HP-5 ms (30 m × 0.25 mm × 0.25 μm; Agilent Technologies). The GS-MS conditions were as follows: start at 70°C, hold for 1 min, increase to 150°C at 10°C min^−1^, 5 min hold, increase at 5°C min^−1^ to 260°C, hold for 15 min, inlet temperature 250°C, sample volume 1 μL (shunt ratio 20), ion source temperature 250°C, ionization mode EI Electron energy 70 eV, carrier gas He (99.999%), carrier air velocity 0.5 mL min^−1^, mass scanning range 35 to 350 m/z, and a solvent delay of 5 min (Zhao et al., 2024b).

To confirm that each chromatograph qualitatively analyzed each component, the retention index of the chromatographic peaks and mass spectrometry information were retrieved and compared through the NIST 14 standard mass spectrometry library peak by GC–MS PostRun Analysis software (Shimadzu, Kyoto, Japan) (Yan et al., 2019). Finally, the relative percentage of each component was calculated using the peak area normalization method.

### 2.4 Endophytic fungi

#### 2.4.1 DNA extraction and PCR amplification

The wood samples were disinfected by immersion in 75% ethanol for 1 min, followed by 3.25% sodium hypochlorite for 3 min, and further immersion in 75% ethanol for 30 s. They were subsequently rinsed three times with sterile distilled water (Yao et al., 2019). The genomic DNA was extracted from the endophytic fungi using a modified CTAB procedure as described by Duong et al. (2006). Each wood sample was ground into a powder with liquid nitrogen. A total of 0.5 g of powder was ground at 45 kHz for 250 s using a FastPrep-24 5G (MP Biomedical, Santa Ana, CA, United States). The ground powder was then transferred to 5 mL of pre-heated (60°C) 2× CTAB extraction buffer and incubated at 65°C for 1 h. The concentration of DNA was determined using a NanoDrop2000 spectrophotometer (Thermo Fisher Scientific, Waltham, MA, United States).

The fungal ITS region was amplified using the primers ITS1F (5′-TCCGTAGGTGAACCTGCGG-3′) and ITS2R (5′-TCCTCCGCTTATTGATATGC-3′) in a 20 μL reaction system with TransStart FastPfu DNA Polymerase in a PCR instrument (ABI GeneAmp^®^ 9,700, Applied Biosystems, Foster City, CA, United States). The PCR amplification conditions were as follows: pre-denaturation at 95°C for 5 min followed by 30 s at 95°C and 30 s at 55°C. The concentrations and amounts used were as follows: the PCR reaction mixture contained 4 μL of 5× FastPfu buffer, 2 μL of 2.5 mM dNTPs, 0.8 μL of forward primer (5 μM), 0.8 μL of reverse primer (5 μM), 0.4 μL of FastPfu polymerase, 10 ng of template DNA, and 2 μL of ddH_2_O. The reaction underwent 25 cycles at 72°C for 45 s followed by a final extension step at 72°C. The PCR product was purified using an AxyPrep DNA Gel Extraction Kit (E.Z.N.A™ Mag-Bind Soil DNA Kit, Omega Bio-tek, Norcross, GA, United States) according to the manufacturer’s instructions. The amplicons were extracted using 2% agarose gel electrophoresis.

#### 2.4.2 Library construction and sequencing

The PCR products were purified and quantified using Qubit^®^ 3.0 (Life Invitrogen, Waltham, MA, United States). A total of 40 different barcode amplicons were mixed equally, and the pooled DNA product was used to create an Illumina pair-end library (Illumina, San Diego, CA, United States) following the Illumina genomic DNA library preparation procedure. The amplicon library was sequenced using an Illumina MiSeq platform (Sangon Biotech (Shanghai) Co., Ltd., Shanghai, China) as previously described.

The raw reads were submitted to the NCBI Sequence Read Archive (SRA) database and assigned Accession Number PRJNA1117010.

#### 2.4.3 Processing of the sequencing data

The raw fastq files were sorted into their respective samples using in-house Perl scripts. This process involved utilizing the barcode sequences for each sample and adhering to specific criteria. First, reads that were 250 bp long and had an average quality score > 20 over a 10 bp sliding window were truncated at the respective site. Secondly, any truncated reads <50 bp were discarded. Barcode matching was performed with precision, allowing for up to two nucleotide mismatches in primer matching. Ambiguous character reads were removed. Sequences with an overlap >10 bp were assembled based on their overlapping sequence. Any reads that could not be assembled were discarded.

The operational taxonomic units (OTUs) were grouped using UPARSE software (version 7.1) from http://drive5.com/uparse/ (accessed on January 8, 2024). A 97% similarity cutoff was applied during the clustering process, and any chimeric sequences were identified and eliminated using UCHIME (v. 4.2.40). A rarefaction analysis was performed using Mothur version 1.21.1 to evaluate the Shannon, Chao1 and evenness diversity indices (Schloss et al., 2009), and an alpha-diversity analysis was conducted based on the UniFrac. The diagram that shows the abundant accumulation of endophytic fungi at the family and genus levels was created using Origin 2021 (OriginLab, Northampton, MA, United States). The β-diversity based on non-metric multidimensional scaling (NMDS) was analyzed using the ‘vegan’ package in R software (version 2.5–6, http://www.r-project.org/), and the significance of clustering was evaluated using a similarity analysis (ANOSIM) (Deng et al., 2021). Additionally, a network diagram to illustrate the endophytic fungal symbiosis and a correlation analysis were generated using the R software package.

### 2.5 Data preprocessing and statistical analysis

All the data were first tested for normality and homogeneity before an analysis of variance (ANOVA) using SPSS 25.0 (IBM Inc., Armonk, NY, United States). The data were also normalized using log_10_ (X + 1) transformations where necessary. The interaction between induction treatment and time was verified using a two-way analysis of variance (ANOVA). A principal coordinate analysis (PCoA) and ANOSIM were performed using the vegan package to visualize and quantify dissimilarities in the potential functions of fungi. All the graphs were created using the Origin Pro2024 (OriginLab Corp., Northampton, MA, United States).

Co-occurrence networks of the fungi were constructed to explore interactions between the species of endophytic fungi at different times of induction. A Spearman correlation matrix was computed using the ‘psych’ package in Rstudio (RStudio, Inc., Boston, MA, United States). Values of r > |0.6| and *p* < 0.05 were set as the thresholds to construct the modular network. Gephi 0.9.1^1^ was used to visualize the fungal networks and calculate the topological properties (Wang et al., 2024). A Mantel test was performed to assess the main drivers that significantly correlated with the aromatic components using the ‘vegan’ package in RStudio, and a correlation heat map was generated using ChiPlot^2^ (accessed on January 10, 2024). The level of statistical significance was set at *p* < 0.05.

## 3 Results and analyses

### 3.1 Relative contents of the aromatic compounds

After the three induction treatments, 69 aromatic components were identified in the *A. sinensis* xylem (Table 2). A total of 41, 37, and 45 aromatic components in the wood samples were induced by the T1, T2, and T3 treatments, respectively. These major components included terpenes, chromones, aromatics, and alkanes. The T1 treatment had the highest concentration of major aromatics, which comprised 82.23% of the total, followed by the T3 treatment with 80.10%. The T2 treatment had the lowest concentration of aromatics at 77.29%.

**Table 2:** Major aromatics of *Aquilaria sinensis* under the different treatments used to induce agarwood.

No.	Components	Odor	Molecular formula	Relative content (%)		
				T1	T2	T3
1	α-Guaiene^△^	Sweet, woody, creamy, pepper flavored	C_15_H_24_	0.34	–	0.76
2	Santalo^△^	Soft sandalwood	C_15_H_24_O	–	2.14	–
3	α-Muurolene^△^	Grassy, floral with orange blossom oil scent	C_15_H_24_	1.46	–	–
4	4-Methylene-1-methyl-2-(2-methyl-1-propen-1-yl)-1-vinyl-cycloheptane^◇^	Aromatic odor	C_14_H_24_	–	2.07	0.74
5	Nootkatone^△^	Citrus flavor	C_15_H_22_O	0.56	–	0.75
6	Hinesol^△^	Pepper, spicy, woody	C_15_H_26_O	–	0.32	–
7	Caryophyllene^△^	Light lilac-like aroma	C_15_H_24_	0.79	–	2.37
8	3-Methyl-(2-pentenyl)-2cyclopenten-1-one^◇^	Celery flavor	C_13_H_16_O_3_	0.49	–	–
9	Zierone^△^	–	C_15_H_22_O	0.49	–	1.05
10	Benzaldehyde^□^	Almond, cherry, and nutty aromas	C_7_H_6_O	1.65	1.79	2.34
11	Limonene^△^	Pleasant, piney, lemony odor	C_10_H_16_	1.23	0.49	0.76
12	3-Methyl-decane^◇^	Aromatic odor	C_11_H_24_	0.57	–	3.24
13	Naphthalene,1,2,3,5,6,7,8,8a-octahydro-1,8a-dimethy^◇^	Citrus-like aromas	C_15_H_24_	–	0.59	–
14	1-Bicyclo-6-dec-4-isopropyl-2,2,3,5,8a-methyl-8H-methylene^◇^	–	C_15_H_24_	–	–	0.3
15	1,5-Dimethyl-2,6-bis(methylene)-Cyclooctane^□^	–	C_12_H_20_	0.49	–	0.44
16	3-Methoxy-4-methyl-2-cyclopenten-1-one^◇^	–	C_7_H_10_O_2_	0.82	–	–
17	2-tert-Butylphenol^◇^	Phenol odor	C_10_H_14_O	–	4.32	–
18	Carvone^△^	Peppermint liquorice Flavor	C_10_H_14_O	–	–	0.34
19	Thymol^△^	Spicy	C_10_H_14_O	1.59	–	1.07
20	4-Phenylbutan-2-one^□^	Floral	C_10_H_12_O	1.89	1.34	2.17
21	(+)-α-Longipinene^△^	Rosin	C_15_H_24_	–	0.64	–
22	Aromadendrene oxide-(1) ^△^	Citrus-like aromas	C_15_H_24_O	–	0.31	–
23	(−)-Aristolene^△^	Spicy	C_15_H_24_	–	–	1.34
24	Longifolene^△^	Sweet woody rose medical fir needle	C_15_H_24_	1.35	2.01	0.73
25	(+)-α-Funebrene^△^	Cedarwood, sandalwood aromas	C_15_H_24_	–	0.34	1.32
26	2-Methyl-5-(1-methylethenyl)-cyclohexanol^△^	Minty, herbal odor	C_10_H_18_O	0.79	–	0.24
27	Methyleugenol^△^	Sweet clove-aniseed aromas	C_11_H_14_O_2_	–	0.76	1.16
28	Longifolenal^△^	Pinewood	C_15_H_24_O	–	–	0.16
29	1,1,4,7-Tetramethyldecahydro-1H-cyclopropa[e]azulene^△^	Flower, magnolia flower aroma	C_14_H_22_O	1.21	–	1.14
30	Isoaromadendrene epoxide^△^	Citrus-like aromas	C_15_H_24_O	1.32	–	0.82
31	Neoclovene^△^	Peppermint odor	C_15_H_24_	0.56	–	0.31
32	Alloaromadendrene^△^	Woody	C_15_H_24_	2.10	3.42	1.23
33	β-Ionone^△^	Warm, woody and with a strong violet aromas	C_13_H_20_O	0.88	–	1.34
34	Epoxide isoheptene^△^	Woody, ester odor	C_16_H_24_O_3_	–	0.64	–
35	Aromandendrene^△^	Sweet, woody	C_15_H_24_	–	0.45	0.74
36	Viridiflorene^△^	Fragrant odor	C_15_H_24_	–	0.25	–
37	γ-Gurjunenepoxide^△^	Sweet citrus, smoky, slightly spicy flavors	C_15_H_24_O	–	1.04	–
38	Isocaryophyllene^△^	Spicy, woody, citrus, camphoreous	C_15_H_24_	0.43	–	0.32
39	Valerena-4,7(11)-diene^△^	Lilac, woody, and spicy aromas	C_15_H_24_	0.64	–	–
40	Cedrol^△^	Cedarwood scented, sweet	C_15_H_26_O	–	0.59	0.32
41	Hexadecane^◇^	Slight hydrocarbon odor	C_16_H_32_	0.71	–	0.11
42	Aromadendrene oxide-(2) ^△^	Orange-like aromas	C_15_H_24_O	1.01	0.56	0.95
43	Isolongifolone^◇^	Pine-like woody	C_15_H_24_O	–	0.31	–
44	Germacrene B^△^	Lemon flavor and fresh aromas	C_15_H_24_	0.73	–	–
45	β-Vatirenene^△^	Orange-like aromas	C_15_H_24_	0.52	–	–
46	Neointermedeol^△^	–	C_15_H_26_O	–	0.54	0.15
47	6-Benzyloxy-3,4-dihydro-4,4-dimethyl-Coumarin^□^	Sweet hay flavor	C_18_H_18_O_3_	1.36	4.4	1.34
48	Tridecanoic acid^◇^	Special fatty acid odor	C_13_H_26_O_2_	6.37	–	2.01
49	Agarospirol^△^	Woody, nutty aromas, burnt	C_15_H_26_O	0.56	–	0.79
50	3-Heptadecanone^◇^	–	C_17_H_34_O	–	1.32	0.21
51	Coniferyl alcohol^△^	Turpentine odor	C_10_H_12_O_3_	5.37	0.19	2.32
52	1,5-Diphenyl-1-Penten-3-one^□^	Spicy	C_17_H_16_O	–	–	0.56
53	5-(2-Thienyl)-4-Pyrimidinamine,^◇^	–	C_8_H_7_N_3_S	2.80	–	-
54	(Z)-13-Docosenamide^◇^	Strong pungent odor	C_22_H_43_NO	–	3.16	–
55	*n*-Hexadecanoic acid^◇^	Slightly waxy fatty	C_16_H_32_O_2_	7.60	11.68	7.4
56	Valerenic acid^△^	Disagreeable odor	C_15_H_22_O_2_	–	0.34	–
57	*trans*-Sinapyl alcohol^△^	Spicy	C_11_H_14_O_4_	2.07	1.63	0.69
58	2-Phenethyl-4H-chromone^○^	Almond, woody and nutty aromas	C_17_H_14_O_2_	–	1.01	1.79
59	Hydroxy-8a,5-dimethyl-3-methylene-3aNaphtho[2,3-b]furan-2-one^◇^	–	C_15_H_20_O_4_	0.75	–	–
60	3,5-Dihydroxy-4-methoxybenzoic acid^◇^	Unique aroma	C_8_H_8_O_5_	0.34	–	3.24
61	2-(4-Methoxyphenethyl) chromone^○^	Almond, woody and nutty aromas	C_18_H_16_O_3_	2.45	–	–
62	Squalene^△^	Pleasant odor	C_30_H_50_	1.32	0.25	–
63	6-Methoxy-2-(2-phenylethyl) chromone^○^	Almond, woody and nutty aromas	C_18_H_16_O_3_	–	2.56	–
64	6,7-Dimethoxy-2-(phenylethyl) chromone^○^	Almond, woody and nutty aromas	C_19_H_18_O_4_	16.89	23.69	22.37
65	6-Methoxy-2-(4-methoxyphenethyl) chromone^○^	Almond, woody and nutty aromas	C_19_H_18_O_4_	1.35	–	3.57
66	6,7-Dimethoxy-2-(2-(4-methoxyphenyl)ethyl) chromone^○^	Almond, woody and nutty aromas	C_19_H_18_O_4_	9.86	4.70	4.52
67	6-Methoxy-2-[2-(4-methoxyphenyl)ethyl]chromone^○^	Almond, woody and nutty aromas	C_20_H_18_O_4_	1.15	1.04	–
68	6,7-Dimethoxy-(4-Methoxyphenethyl) chromone^○^	Almond, woody and nutty aromas	C_20_H_20_O_5_	–	0.65	1.32
69	N-4-benzyloxyphenyl isobutyrylacetamide^◇^	–	C_19_H_21_NO_3_	–	0.69	1.65
The relative content of terpenes (%)				27.10a	16.68b	25.47b
The relative content of chromones (%)				31.70a	33.65a	33.57a
The relative content of aromatics (%)				3.54a	3.13a	5.07a
The relative content of alkanes (%)				19.89b	23.83a	15.99c
The total relative content of aromatics (%)				82.23a	77.29b	80.10ab

△, Terpenes; ○, chromones; □, aromatics; ◇, alkanes. Different lowercase letters indicate significant differences among the treatments (*P* < 0.05).

This study found that the relative content of aromatics varied depending on the induction treatment. Terpenes had a higher relative content of 27.10 and 25.47% under the T1 and T3 treatments, respectively, which was significantly higher than that of the T2 treatment. Under the T1 and T2 treatments, there were significantly more alkanes at 19.89 and 23.83%, respectively, compared to the T3 treatment. However, the absence of chromones and aromatic components was found to correlate with its different induction treatments.

The primary terpenes in the wood samples from the T1 treatment contained coniferyl alcohol (C_10_H_12_O_3_, 5.37%) and alloaromadendrene (C_15_H_24_, 2.10%). Moreover, the T2 treatment had higher levels of alloaromadendrene (C_15_H_24_, 3.42%), santalol (C_15_H_24_O, 2.14%), and longifolene (C_15_H_24_, 2.01%). The wood samples from the T3 treatment contained caryophyllene (C_15_H_24_, 2.37%), coniferyl alcohol (C_10_H_12_O_3_, 2.32%), and β-ionone (C_13_H_20_O, 1.34%). The most prevalent chromones were 6, 7-dimethoxy-2-(phenylethyl) chromone (C_19_H_18_O_4_, 16.89–23.69%) and 6,7-dimethoxy-2-(2-(4-methoxyphenyl)ethyl) chromone (C_19_H_18_O_4_, 4.52–9.86%) in the wood samples induced by the T1, T2, and T3 treatments, respectively. The wood samples induced by the T1, T2, and T3 treatments had the highest relative content of aromatics in 6-benzyloxy-3,4-dihydro-4, 4-dimethyl-coumarin (C_18_H_18_O_3_,1.34–4.40%), benzaldehyde (C_7_H_6_O, 1.65–2.34%), and 4-phenylbutan-2-one (C_10_H_12_O, 1.34–2.17%), respectively. The highest relative content of alkanes was found in *n*-hexadecanoic acid (C_16_H_32_O_2_, 7.40–11.68%) in the wood samples induced by the T1, T2, and T3 treatments.

### 3.2 Endophytic fungi

#### 3.2.1 Characterization of the sequencing data

**Figure 2:**
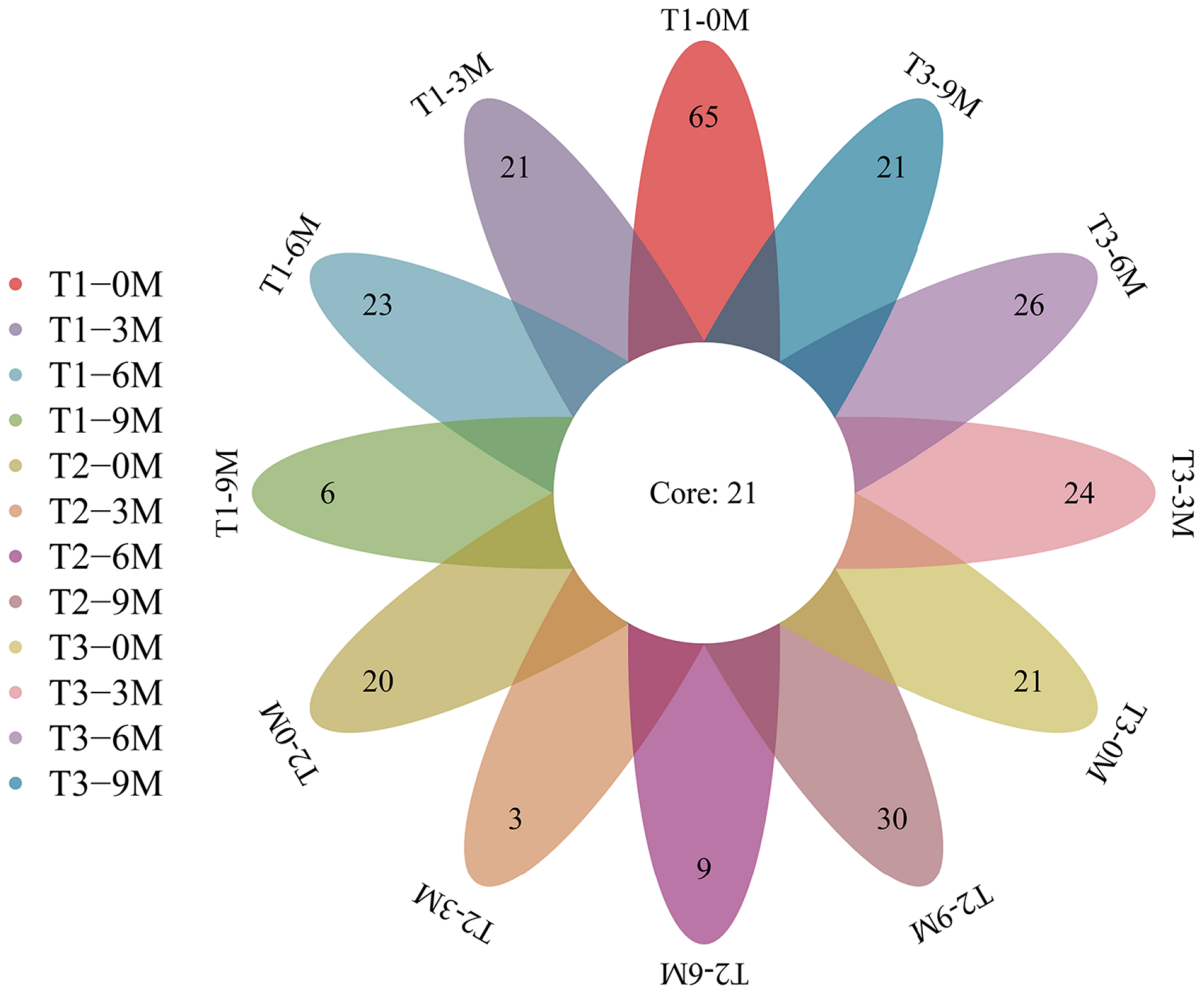
Venn diagram of the OTU for each sample, 0 M, 3 M, 6 M, and 9 M represent pre-induction, the third month, the sixth month and the ninth month after artificial induction, respectively.

A total of 7,147,492 high-quality sequences were obtained from 40 wood block samples from the *A. sinensis* xylem to analyze the diversity of endophytic fungi. The sequences were clustered based on 97% sequence similarity, which resulted in the generation of 494 OTUs. Of these, 115, 62, and 92 were specific to the T1, T2, and T3 treatments, respectively, while 21 OTUs were shared among the three treatments (Figure 2). The number of T1 treatment-specific OTUs decreased when the period of induction was prolonged. The highest number of OTUs was observed in the 0 M treatment, while the lowest was observed in the 9 M treatment. The number of T2 treatment-specific OTUs decreased and then increased. The 9 M treatment contained the highest number of OTUs. The number of OTUs specific to the T3 treatment fluctuated. The most OTUs were observed in the 6 M treatment. The number of OTUs increased and reached a peak in the 9 M treatment. The number of OTUs fluctuated in T3, with the highest number observed in the 6 M treatment.

**Figure 3:**
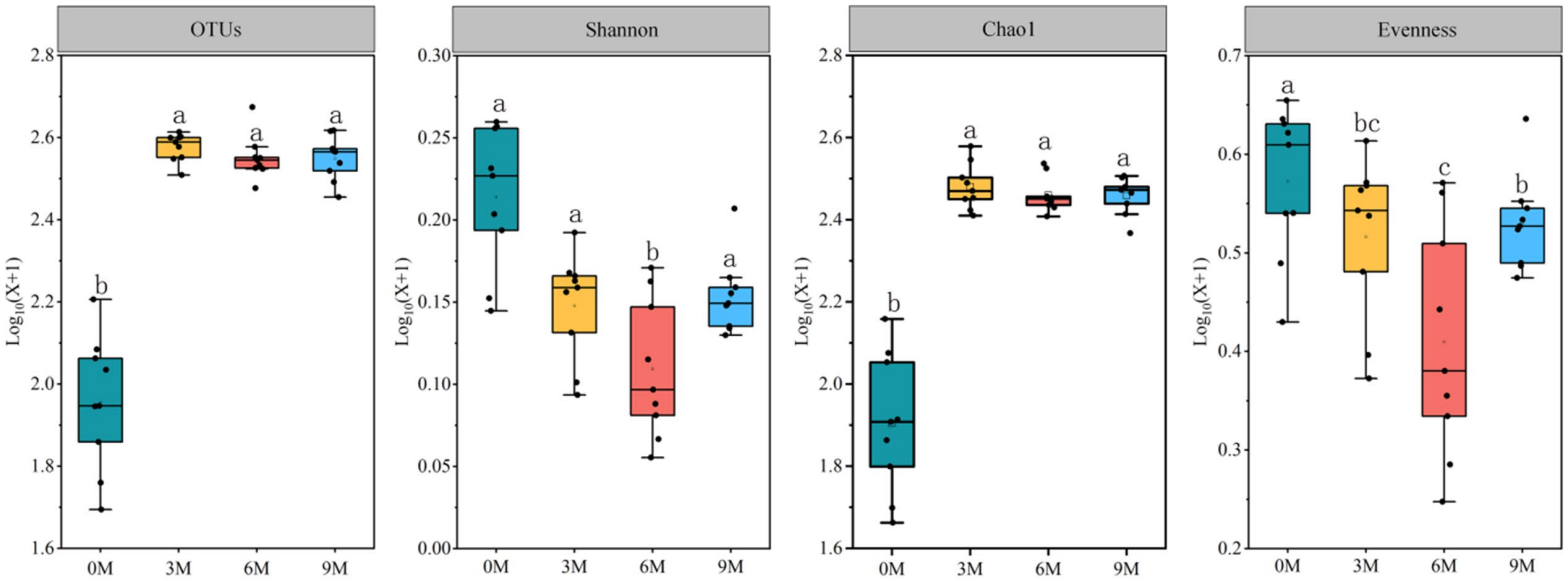
Changes in the alpha-diversity of the endophytic fungal communities in the *Aquilaria sinensis* xylem at different times of induction. 0 M, 3 M, 6 M, and 9 M represent pre-induction, the third month, the sixth month, and the ninth month after artificial induction, respectively. Different letters indicate significant differences between the induction time groups (*p* < 0.05).

The alpha-diversity analysis (Figure 3, Supplementary Tables S1–S3) revealed a significant difference (*p* < 0.05) in the endophytic fungal Shannon index between the T1, T2, and T3 induction treatments. Additionally, no significant difference (*p* > 0.05) was observed in the numbers of sequences and OTUs and the Chao1 and the Palou evenness indices. There were highly significant differences (*p* < 0.01) in the number of OTUs and the Shannon, Chao1, and the Palou evenness indices in the endophytic fungal community at different sampling timepoints for each induction treatment with no significant differences in sequence numbers. There was no significant difference in the alpha-diversity of the endophytic fungal communities for the interaction of each induction treatment and different sampling times. The results demonstrated that there was a more pronounced impact on the different sampling times on the richness and diversity of endophytic fungal communities in the *A. sinensis* xylem than that of the induction treatments.

#### 3.2.2 Composition of the endophytic fungal community

**Figure 4:**
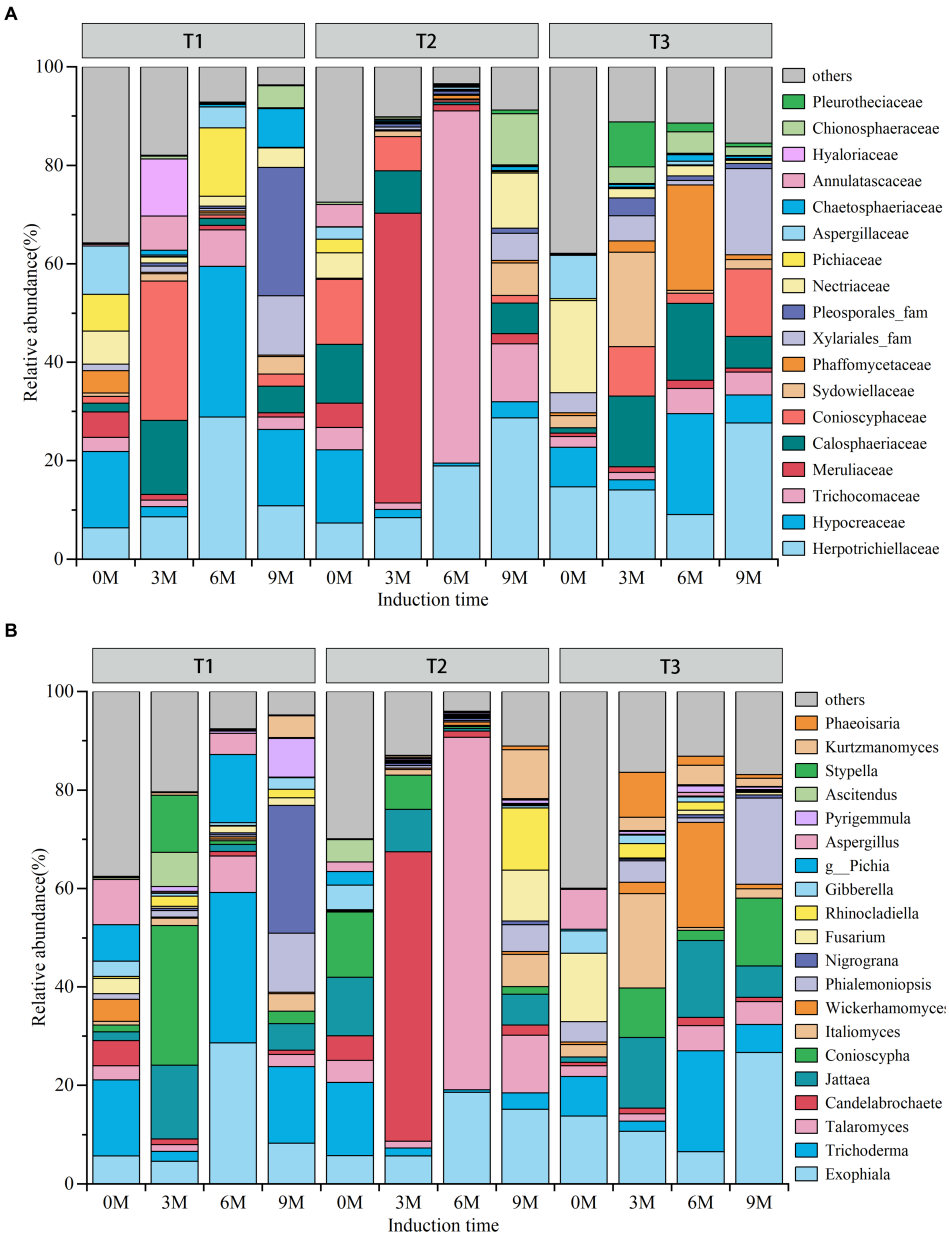
**(A)** Family, **(B)** genus. 0 M, 3 M, 6 M, and 9 M represent pre-induction, the third month, the sixth month, and the ninth month after artificial induction, respectively.

The results of the species annotation of OTUs demonstrated that the endophytic fungi were primarily composed of 18 dominant families and 20 dominant genera (Figures 4A,B). As time progressed, the relative abundance of endophytic fungi in the dominant families and genera increased in the T1 induction treatments, while they increased and then decreased in the treatments induced by T2 and T3. In contrast, the abundance of non-dominant families exhibited an opposite trend.

Among the endophytic fungal dominant families, the highest relative abundance in the T1 induction treatment was 6 M Hypocreaceae (30.59%) and Herpotrichiellaceae (28.89%), followed by 3 M Conioscyphaceae (28.38%) and 9 M Pleosporales_fam (26.02%) and Hypocreaceae (15.51%). The highest relative abundance found in the T2 treatment was 6 M Trichocomaceae (71%). The most prevalent taxa were 56% followed by 3 M Meruliaceae (58.82%) and 9 M Herpotrichiellaceae (28.67%). The T3 induction treatment was 9 M Herpotrichiellaceae (27.64%) followed by 6 M Phaffomycetaceae (21.40%), Hypocreaceae (20.50%) and Calosphaeriaceae (15.67%) and 3 M Sydowiellaceae (19.20%) among others.

Among the dominant genera of endophytic fungi, the highest relative abundance in the T1 induction treatment was 6 M *Trichoderma* (30.59%) and *Exophiala* (28.61%) followed by 3 M Conioscyphaceae (28.38%) and 9 M *Nigrograna* (25.92%). In the T2 induction treatment, 6 M *Talaromyces* (75.56%) was the most abundant followed by 3 M *Candelabrochaete* (58.82%) and 9 M *Exophiala* (15.16%). The most abundant genera in the T3 induction treatments were 9 M *Exophiala* (26.70%) followed by 6 M *Wickerhamomyces* (21.39%), *Trichoderma* (20.50%) and *Jattaea* (15.67%) and 3 M *Italiomyces* (19.20%).

**Figure 5:**
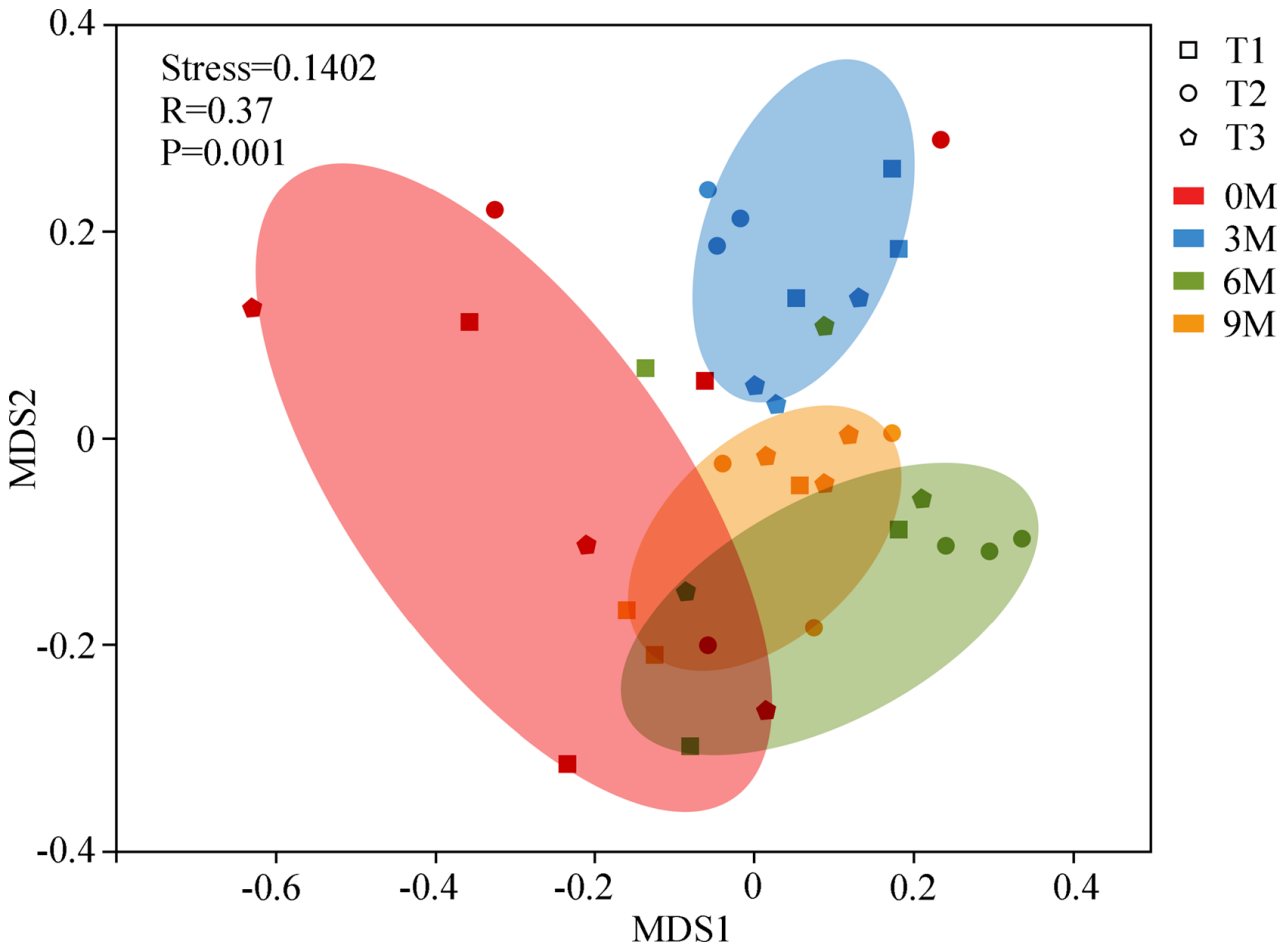
Non-metric multidimensional scaling analysis (NMDS) of the endophytic fungal communities and analysis of similarity (ANOSIM). 0 M, 3 M, 6 M, and 9 M represent pre-induction, the third month, the sixth month, and the ninth month after artificial induction, respectively.

The endophytic fungal community structures between the groups of different induction time were compared using an NMDS based on the Bray-Curtis distances (Figure 5) and an ANOSIM analysis based on the Weighted UniFrac distances. The NMDS analysis illustrated that the fungal β-diversity clearly clustered into four groups according to the induction time. The ANOSIM also demonstrated significant differences in the structural changes in the endophytic fungi across the sampling times for each induction treatment (*R* = 0.37; *p* = 0.001), which suggested that there was a more pronounced differentiation in the endophytic fungal community structure between the individual induction treatments as the time of induction increased.

#### 3.2.3 Endophytic fungal dominant family and genus

**Figure 6:**
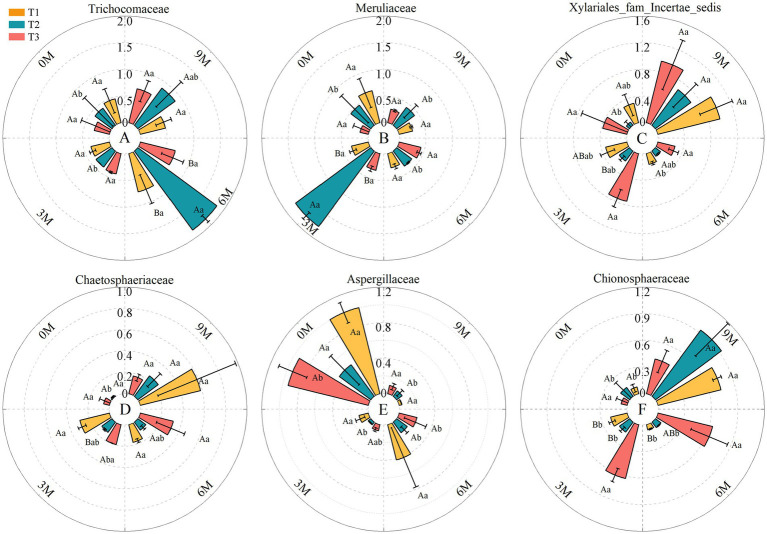
ANOVA for fungi of the dominant family. The relative abundance of each indicator in the above figure was converted to log10(X + 1) standards. 0 M, 3 M, 6 M, and 9 M represent pre-induction, the third month, the sixth month, and the ninth month after artificial induction, respectively. The different treatments are presented in upper case, while the same treatment at different times is presented in lower case.

An ANOVA showed that among the six dominant families of endophytic fungi in each induction treatment, the relative abundance of Aspergillaceae fluctuated as the time of induction was prolonged, whereas the relative abundance of Meruliaceae and Trichocomaceae increased and then decreased. The former reached a peak at 3 M and the latter at 6 M, and the relative abundances of Xylariales_fam, Chaetosphaeriaceae and Chionosphaeraceae increased, which indicated that they are the important predominant families of fungi in the formation of aromatic components in the *A. sinensis* xylem (Figure 6).

**Figure 7:**
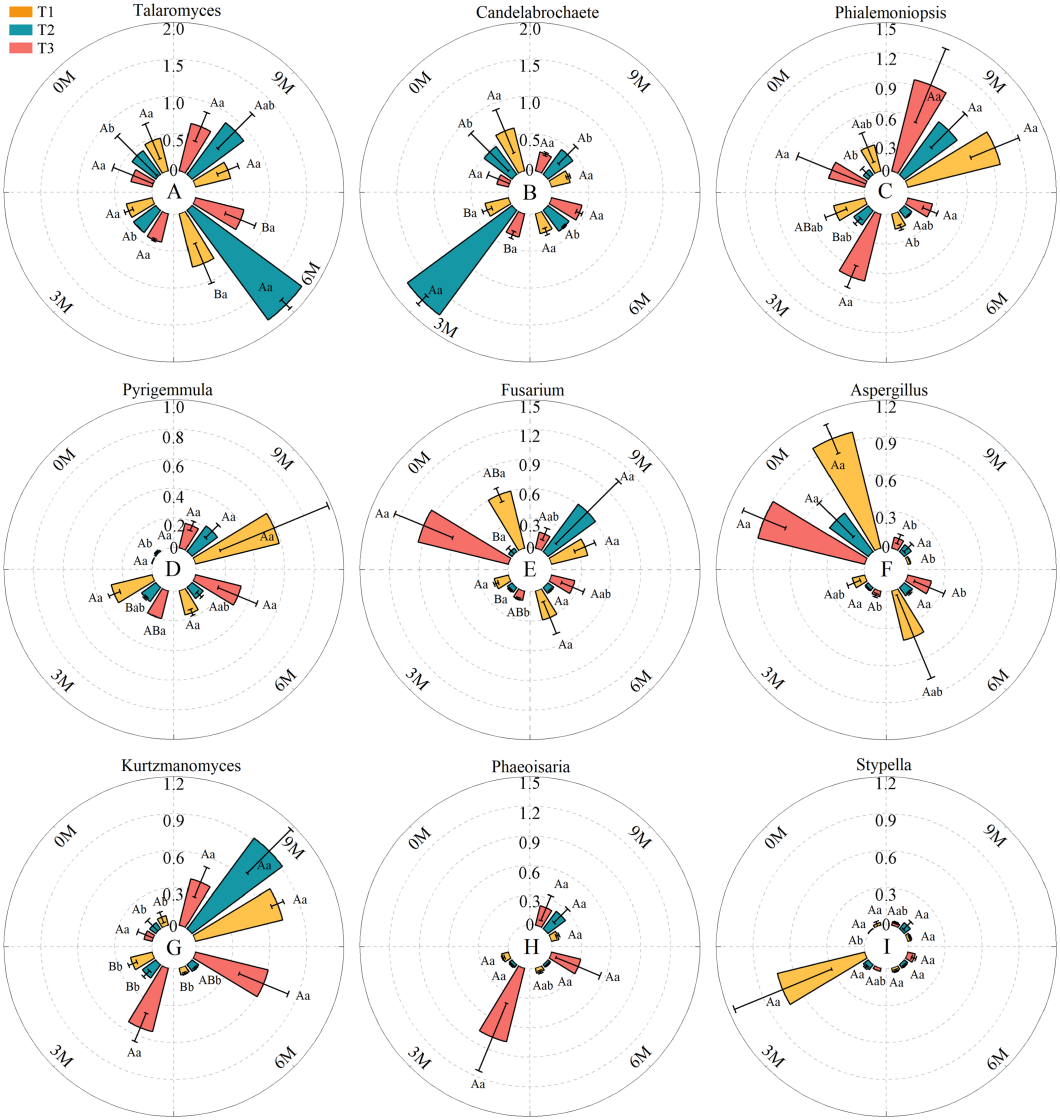
ANOVA for the dominant genera of fungi with different treatments at the same time presented in uppercase, and the same treatments at different times presented in lowercase. The relative abundance of each indicator in this figure was converted to log10(X + 1) standards. 0 M, 3 M, 6 M, and 9 M represent pre-induction, the third month, the sixth month, and the ninth month after artificial induction, respectively. Different treatments are presented in uppercase, while the same treatment at different times is presented in lowercase.

A higher resolution among the nine dominant genera endophytic fungi showed that the relative abundances of *Aspergillus* and *Fusarium* fluctuated as the induction time was prolonged, and the relative abundances of *Talaromyces, Candelabrochaete*, *Phaeoisaria*, and *Stypella* first increased and then decreased and peaked successively at 3 M. In contrast, the relative abundances of *Phialemoniopsis, Pyrigemmula* and *Kurtzmanomyce* increased, which led to competition for the dominant genera of endophytic fungi in the *A. sinensis* xylem (Figure 7).

**Figure 8:**
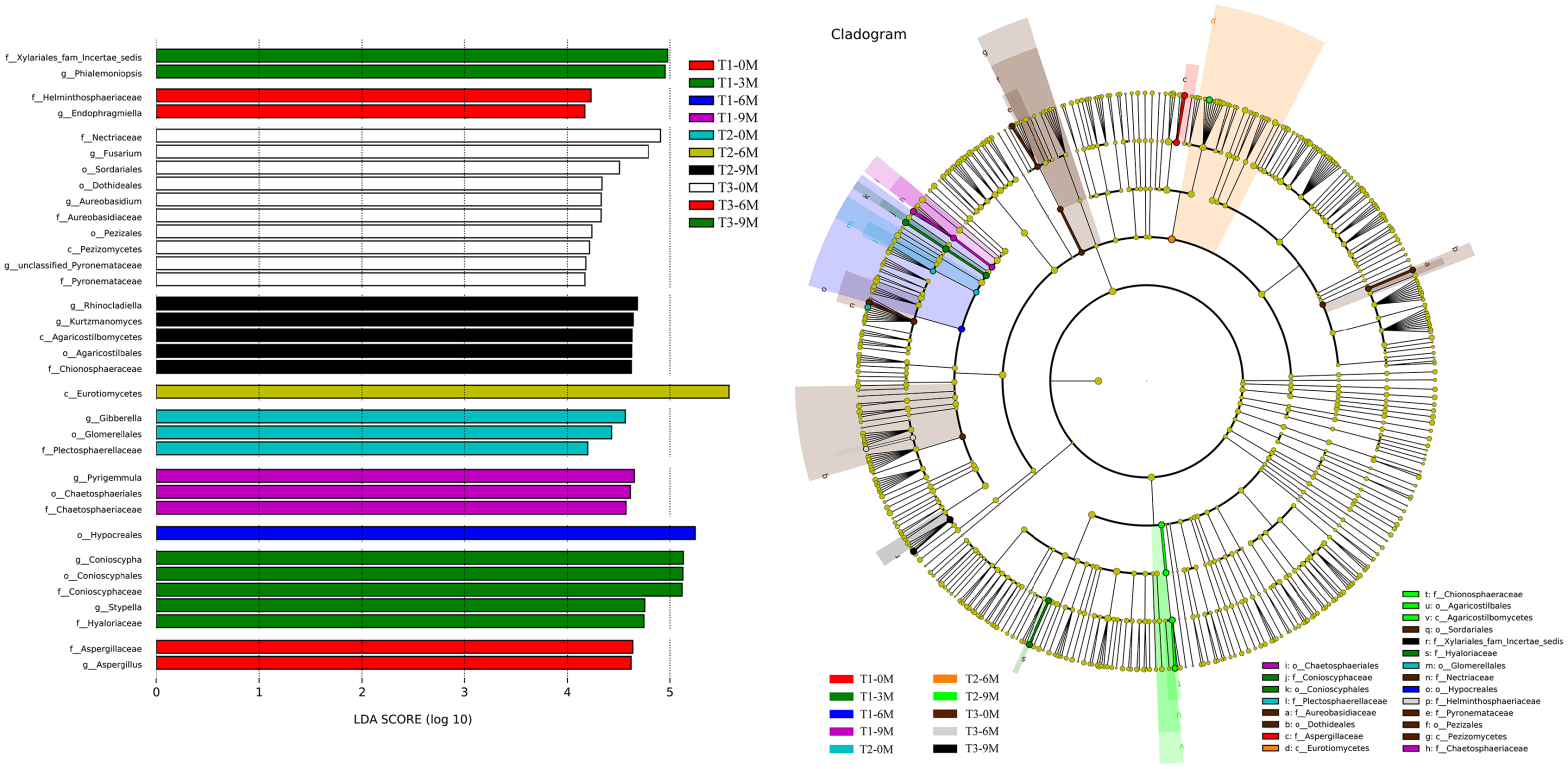
Differential analysis of the LEfSe of the endophytic fungal communities, 0 M, 3 M, 6 M, and 9 M represent pre-induction, the third month, the sixth month, and the ninth month after artificial induction, respectively.

The Linear discriminant analysis Effect Size (LEfSe) difference analysis of the endophytic fungal histograms of the Linear discriminant analysis (LDA) value distribution and species evolutionary branching diagrams showed the groups that were significantly different between the T1-0 M, T1-3 M, T1-6 M, T1-9 M, T2-0 M, T2-6 M, T2-9 M, T3-0 M, T3-6 M and T3-9 M groups (Figure 8). Aspergillaceae, *Aspergillus*, *Endophragmiella* and Helminthosphaeriaceae were the groups that varied the most between T1-0 M and T3-6 M. Xylariales_fam Incertae sedis, *Phialemoniopsis*, *Conioscypha*, *Conioscyphales*, *Conioscyphacea*, Stypella, and Hyaloriaceae were the groups that were primarily responsible for the difference between the T1-3 M and T3-9 M treatments. Hypocreales was the group that had the most significant effect on the difference between T1-6 M, while Pyrigemmula, Chaetosphaeriales and Chaetosphaeriaceae were the groups that were primarily differentiated in T1-9 M. *Gibberella*, Glomerellales and Plectosphaerellaceae were the groups that were primarily differentiated in T2-0 M, while Eurotiomycetes was the group that was primarily differentiated in T2-6 M. *Rhinocladiella, Kurtzmanomyces, Agaricostilbomycetes, Agaricostilbales,* and Chionosphaeraceae were the predominantly differentiated groups in T2-9 M. Nectriaceae, *Fusarium, Sordariales, Dothideales, Aureobasidium, Aureobasidiaceae, Pezizales*, Pezizomycetes, unclassified_Pyronemataceae and Pyronemataceae were the microflora that played a major difference in T3-0 M. These results indicated that there was significant differential microflora in the xylem endophytic fungal communities of *A. sinensis* at different sampling times for each induction treatment.

#### 3.2.4 Co-occurrence network of the endophytic fungal communities

**Figure 9:**
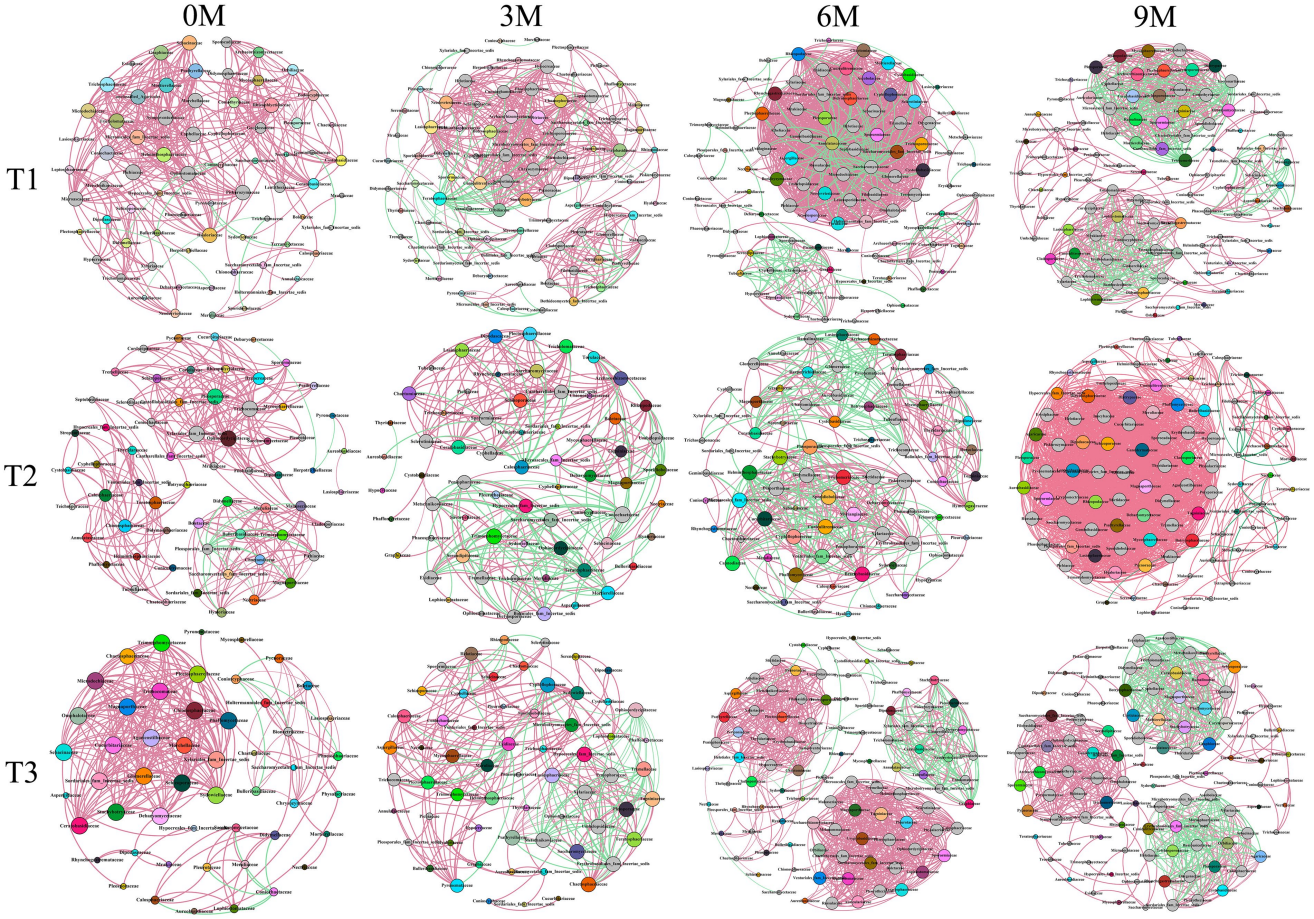
Co-occurrence networks of the endophytic fungi communities in the three induction treatments at different induction times based on the family level and correlation analysis. The nodes in network are colored by family and the size of each node is proportional to the number of connections. The red and green edges represented the positive and negative correlation, respectively (Pearson, *p* < 0.05). The edge thickness primarily depends on the correlation coefficient. 0 M, 3 M, 6 M, and 9 M represent pre-induction, the third month, the sixth month, and the ninth month after artificial induction, respectively.

A co-occurrence network relationship graph at the endophytic fungal family level was constructed based on the wood block samples at different sampling times for each induction treatment as shown in Figure 9 and Supplementary Table S4. A total of 12 endophytic fungal networks were constructed, with the T1 induction treatment consisting of 336, 445, 1,129, and 635 edges, which connected 71, 89, 99, and 99 nodes, respectively. The treatment induced by T2 consisted of 286, 269, 431, and 1,579 edges, which connected 63, 66, 77, and 97 nodes, respectively. The T3 induced treatment consisted of 272, 268, 742, and 656 edges, that connected 51, 62, 97, and 101 nodes, respectively.

The results of the T1 induction treatment indicated that the 0 M endophytic fungal high connectivity nodes were Hyaloriaceae and Microascaceae among others. The 3 M high connectivity nodes were Ceratobasidiaceae and Choanephoraceae among others, and the 6 M high connectivity nodes were Annulatascaceae and Didymosphaeriaceae among others. Additionally, the 9 M high connectivity nodes were observed for Cyphellophoraceae and Helotiaceae among others. In the context of the T2 induction treatment, the 0 M high connectivity nodes for the endophytic fungi were Ophiocordycipitaceae and Pleosporaceae among others, while the 3 M high connectivity nodes were Calosphaeriaceae and Coniochaetaceae among others. The 6 M high connectivity nodes were Brachybasidiaceae and Pleosporaceae among others, and the 9 M high connectivity nodes were Botryosphaeriaceae and Ganodermataceae among others. In the context of the T3 induction treatment, the 0 M high connectivity nodes of endophytic fungi were Agaricostilbaceae and Chionosphaeraceae among others. The 3 M high connectivity nodes were Archaeorhizomycetaceae and Corynesporascaceae among others, and the 6 M high connectivity nodes were Cryptobasidiaceae and Magnaporthaceae. Finally, the 9 M high connectivity nodes were Annulatascaceae and Ceratobasidiaceae among others.

**Figure 10:**
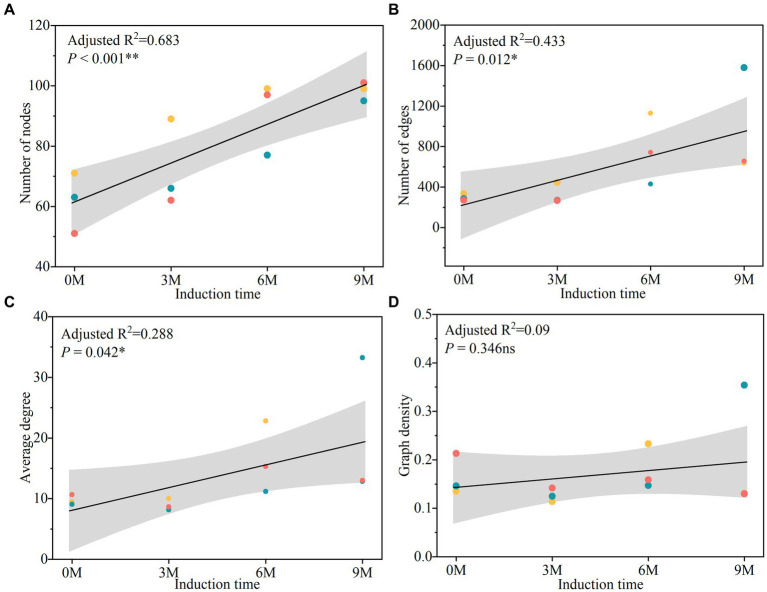
Linear regression analyses of the network topology change with induction times, including node numbers **(A)**, edge numbers **(B)**, average degree **(C)** and graph density **(D)**. 0 M, 3 M, 6 M, and 9 M represent pre-induction, the third month, the sixth month, and the ninth month after artificial induction, respectively.

The topological indices of the endophytic fungal co-occurrence network (Figure 10 and Supplementary Table S4) indicated that the nodes tended to increase in parallel with the time of induction, while in most edges, the average degree and graph density tended to increase initially and then decrease. The results indicated that the endophytic fungi were more closely related to each other, and the structure of the community network became more complex with the extension of time between the various induction treatments.

### 3.3 Relation between the aromatic components and fungal community diversity

**Figure 11:**
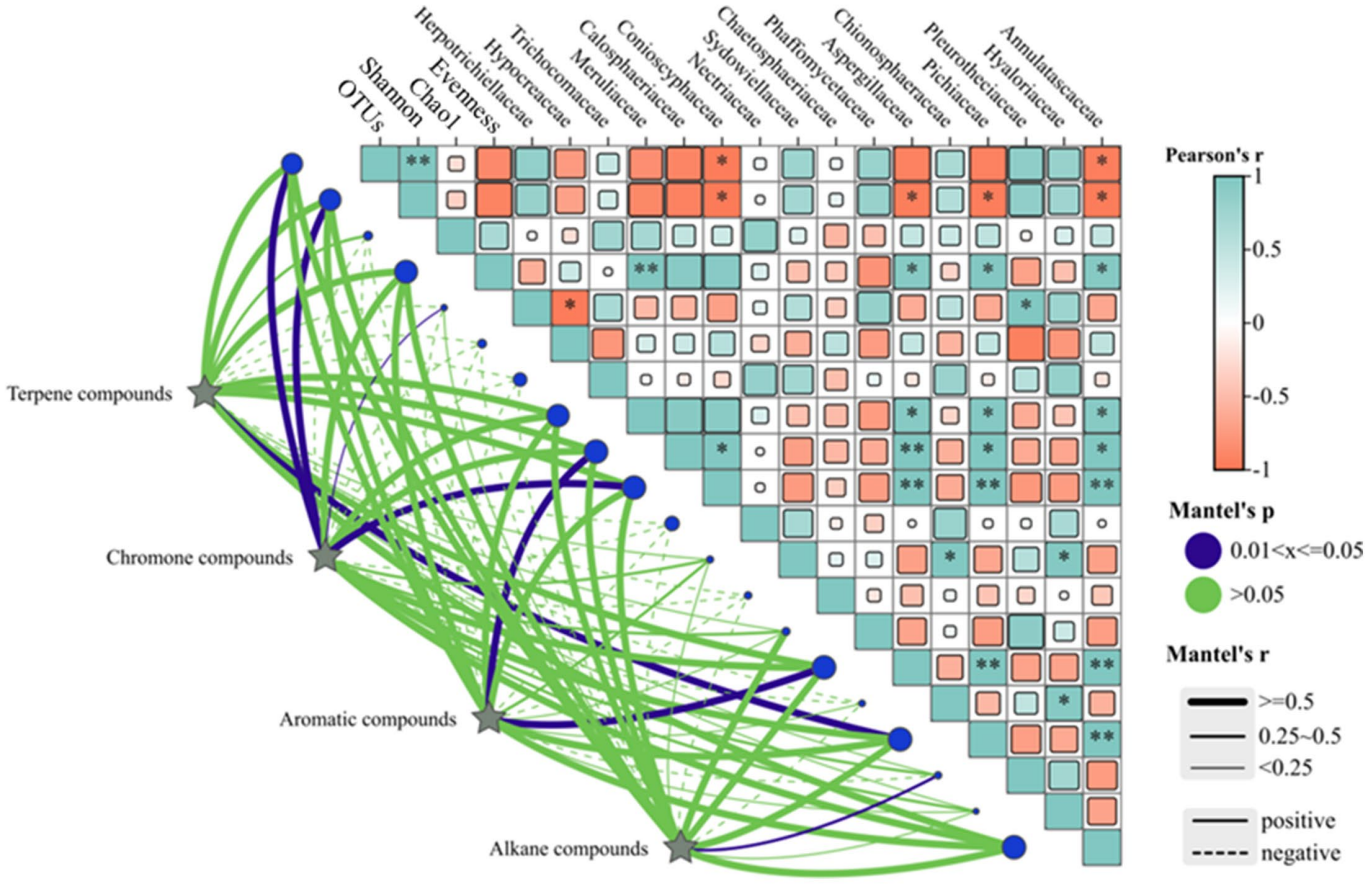
Correlation analyses of the aromatic components with the endophytic fungi of the main dominant families and their community diversity.

A combined plot analysis of the correlation between the xylem aromatic components and diversity of the endophytic fungal community and endophytic fungi of the dominant genera in *A. sinensis* showed that the main volatile terpene compounds significantly negatively correlated with the Pielou evenness index (*p* < 0.05) and did not correlate with the other endophytic fungal community diversity indices (*p* > 0.05) (Figure 11). The chromones significantly positively correlated with the OTUs and Shannon indices, while the aromatics did not significantly correlate with the OTUs, Shannon, Chao1 and Evenness indices. The alkanes significantly negatively correlated with the Shannon index and significantly positively correlated with the Pielou evenness index.

The principal volatile terpenes significantly negatively correlated with Meruliaceae, whereas no significant correlation was observed with the other endophytic fungi that were members of the dominant genera. The chromones were also found to significantly negatively correlate (*p* < 0.01) with Aspergillaceae, Annulatascaceae and Conioscyphaceae and not significantly correlate with Calosphaeriaceae and Pichiaceae. The chromones negatively correlated with Aspergillaceae, Annulatascaceae and Conioscyphaceae (*p* < 0. 01) and significantly correlated with Calosphaeriaceae and Pichiaceae. The aromatics did not significantly correlate with any of the dominant genera of endophytes. The alkanes did not significantly correlate with Aspergillaceae, Annulatascaceae, Conioscyphaceae, Conioscyphaceae and Conioscyphaceae, Aspergillaceae, Annulatascaceae, Conioscyphaceae, Meruliaceae and Pichiaceae, However, there was a correlation with the other dominant genera of endophytic fungi.

## 4 Discussion

### 4.1 Effect of the different induction treatments on the formation of aromatics in the *A. sinensis* xylem

The results of the GC–MS analysis revealed that the aromatics present in the xylem of the three treated *A. sinensis* were primarily chromones (31.70–33.65%), terpenes (16.68–27.10%), alkanes (15.99–23.83%), and aromatics (3.13–5.07%), which are basically consistent with the main chemical components of natural agarwood. Previous studies have indicated that chromones, terpenes, and aromatics are the characteristic aromatic compounds of *A. sinensis* that are related to the quality of agarwood.

Studies have shown that there are between 10 and 15% of terpenoids in the agarwood obtained from the induction treatments with exogenous substances. The main components of the induction of *A. sinensis* using *Fusarium sclerotiorum* and *Sclerotinia sclerotiorum* included terpenes, such as epoxidized isogeranocene, oxidized geranocene, sedum spirolol, guaiacol, gimarone, and leucovorinol, with a total relative content of 55.70% (Chen et al., 2018). The terpenes (sedum spirol, sedum furan, albizia aldehyde and spearmint alcohol) and aromatics (benzylacetone and *p*-methoxybenzylacetone) obtained by using biological inoculation, flaming and chemical stimulation treatments are the main aromatics of agarwood (Meier et al., 2003; Zhang et al., 2014), and all these results differed from the conclusions of this study. The results of this study also differ from the findings that 2-(2-(4-methoxyphenyl)ethyl) chromone, sedum spirol, guaiacodiene-15-aldehyde, dihydrocarrageenone, and cedrol are the main aromatic components of *A. malaccensis* (Tibpromma et al., 2021). It is apparent that the differences in the chemical substances and relative content of the main aromatic components in agarwood may be closely related to the species of *Aquilaria*, method and duration of agarwood formation, and genetic factors (Subasinghe et al., 2019; Xiang et al., 2020).

During the formation of aromatic components in *Aquilaria* plants, chromones, benzaldehyde and other aromatics with almond, woody and nutty aromas are among the most strongly aromatic active components, which is similar to the results of the study of the volatile aromatic components of *A. malaccensis, A. subintegra* and *A. crassna*; these are the main aromatic components of agarwood (Pripdeevech et al., 2011). In addition, some aromatics, such as limonene, 4-phenylbutan-2-one, longifolene, alloaromadendrene, aromadendrene oxide-(2), and *n*-hexadecanoic acid, which are characteristic aromatics of agarwood, largely contribute to the aroma. α-Muurolene, isoaromadendrene epoxide, caryophyllene, aromadendrene oxide-(1), valerena-4,7(11)-diene, and tridecanoic acid also contribute to enhancing the aromatic odors of agarwood, with a sweet fruity scent, sweet fruity, floral and herbal aromas that are attributed to the presence of these components. α-Guaiene, methyleugenol, aromandendrene and γ-gurjunenepoxide are the main sources of the sweet aroma, which suggests that the agarwood is derived from a balance between the odors of different volatile aromatic components.

### 4.2 Effect of the different induction treatments on the endophytic fungi in the *A. sinensis* xylem

Although endophytic fungi are involved in the growth, development, and accumulation of secondary metabolites in their host plants, more than 90% of the endophytic fungi in the natural environment cannot be cultured (Huang et al., 2023). This suggests that the tissue isolation methods do not fully reflect the diversity and community structure of endophytic fungi. Therefore, we collected wood block samples for endophytic fungal ITS sequencing during the process of using exogenous substances to induce the formation of aromatic components in the *A. sinensis* xylem. Since there may be some similarity in the physiological state of individuals in stands of the same age, basically the same numbers of OTUs were counted in common.

In this study, the diversity and richness of the endophytic fungal community were basically insignificant among the treatments, which may be related to the comparable growth environment conditions of *A. sinensis* in the study site. The community structure of fungi in *A. sinensis* within Hainan Province is believed to be similar, and the fungal community structure of *A. sinensis* in Guangdong Province is similar. However, there are significant differences in the fungal composition between the agarwood produced in Guangdong and Hainan, China (Chen et al., 2019). Moreover, with the passage of time, there were significant changes in the predominant endophytic fungal population among the different treatments, which contributed to the pronounced discrepancies in their diversity indices. In addition, the endophytic fungi in the T1 and T3 treatments were more diverse than those in the T2 treatment. With regard to the endophytic fungal community structure, the dominant genera in the T1 treatment were *Trichoderma, Exophiala,* Conioscyphaceae and *Nigrograna*. In the T2 treatment, the dominant genera were *Talaromyces, Candelabrochaete* and *Exophiala*. In the T3 treatment, the four genera that were the most abundant microflora were *Wickerhamomyces, Trichoderma, Jattaea* and *Italiomyces*. Researchers have suggested that there are also significant differences in the abundance of the endophytic fungal communities in the samples of wood blocks from different heights of the *A. sinensis* xylem (Liu et al., 2019). The dominant genera of the endophytic fungi induced by the various treatments of *A. sinensis* in this study differed from those in other studies that identified *Lasiodiplodia, Trichoderma*, and *Phaeoacremonium* (Liu et al., 2022). There are also differences in the dominant genera of endophytic fungi between *A. malaccensis* grown in Malaysia and India with *Fusarium* and *Xylaria* isolates, respectively (Mohamed et al., 2010; Chhipa and Aushik, 2017; Ramli et al., 2022). These results are inconsistent with the conclusions of this study, and the reasons for this may be related to differences in the environment, stand age, tree species, and treatment methods at the study site.

A review of the previous research on this topic revealed that *Exophiala candelabrochaetehe*, *Nigrograna* and *Wickerhamomyces* (Ziegler, 1968; Li et al., 2022; Xiaoning et al., 2022; Fang et al., 2024) are the predominant genera of endophytic fungi commonly found in *Aquilaria* plants. This finding is generally consistent with the results of this study. In addition, *Trichoderma* produces dark green mold on the surfaces of the damaged stems of *A. sinensis*, tomato (*Solanum lycopersicum*) and *Arabidopsis thaliana*. It then infects the stem of the host plant, which results in alterations to its growth, development and accumulation of secondary metabolites (Carillo et al., 2020; Rebolledo-Prudencio et al., 2021; Zhao et al., 2024b). Some fungi, such as Conioscyphaceae, *Talaromyces, Jattaea*, and *Italiomyces*, cause the wood to decay in trees (Dayarathne et al., 2017; Senanayake et al., 2017; Varriale et al., 2018). This process activates the defense responses of *Aquilaria* plants, which biosynthesize secondary metabolites with volatile aromatic odors. These metabolites prevent or delay the proliferation of fungi and resist infection with harmful pathogens. The aromatics that are contained in the oil in the *Aquilaria* xylem are referred to as agarwood. This suggests that the formation of aromatics in the *Aquilaria* xylem is closely related to endophytic fungi and may be the result of the joint action of multiple endophytic fungi.

A correlation analysis identified a negative correlation between Hypocreaceae and Herpotrichiellaceae (Figure 11), which suggests the potential for antagonistic effects between different fungal communities. When the time of induction was prolonged, the dominant endophytic fungi in the *A. sinensis* xylem evolved from the Hypocreaceae to Herotrichiellaceae, *Trichoderma*, Conioschyphaceae, and Pleurothecaceae among others. The decrease in abundance of the former weakened the antagonistic effect between the latter. This suggests that the *A. sinensis* xylem is stimulated by exogenous substances or undergoes changes during its growth process, which results in a change in the originally stable microbial community structure. Changes in the antagonistic, symbiotic, and competitive relationships between the changes in endophytic fungi benefit the growth of some endophytic fungal communities, which may then develop into the dominant microbial communities that damage plants. This may be one of the important reasons behind the promotion of the formation of aromatics in the *A. sinensis* xylem.

### 4.3 Aromatic outcomes of the interaction with endophytic fungi

In this study, 9 months of treatment with different types of inducers led to the observation of significant changes in the interaction among aromatic components, fungal components, and diversity during the formation of woody aromatic components in *A. sinensis*. It has been confirmed that the prevalence of endophytic fungi is closely related to the formation of agarwood and may be the result of the combined action of multiple fungi. Some researchers have also suggested that although the types and numbers of endophytic fungi decrease during the formation of agarwood, there is an increase in the number of dominant populations (Li et al., 2024). Caryophyllene and 2-(2-phenylethyl) chromone isolated from agarwood have antibacterial properties (Dahham et al., 2015; Lei et al., 2018). This indicates that under the long-term action of multiple types of endophytic fungi, *A. sinensis* produces secondary metabolites, including aromatic components, which can enhance its stress resistance and further verify the close relationship between the diversity of endophytic fungi and agarwood formation.

Terpenes and chromones are among the most important characteristic aromatic components of agarwood (Shivanand et al., 2022). Fungi act as elicitors to induce defense responses in *A. sinensis* (Zhao et al., 2024a,b), and plant hormones, such as jasmonic acid (JA), play a key signaling role. Studies have also shown that multiple endophytic fungi can promote the biosynthesis of terpenes through the mevalonic acid (MVA) pathway by modulating the concentrations of plant hormones (Gastaldo et al., 2019; Kou et al., 2021). One of the biosynthetic pathways of chromones is thought to involve the acetate metabolism pathway of the condensation of five malonyl coenzyme A molecules, the biosynthesis of 5, 7-dihydroxy-4-methylchromone by polyketide synthase, and the production of 2-(2-phenylethyl) chromone under the catalysis of cyclooxygenase, which catalyzes the biochemical reaction of the chromone matrices using hydroxylases or *O*-methyltransferases among other enzymes, to form the chromones (Amen et al., 2021). Secondly, 2-(2-phenylethyl) chromone was produced by the decarboxylation of malonyl coenzyme A, which was catalyzed by polyketide synthase to produce acetyl coenzyme A as a starting substrate, and hydroxylase and *O*-methyltransferase then catalyzed the production of chromones from the chromone mother nucleus (Liao et al., 2018).

In the study of the interaction between *A. malaccensis* and Fusarium, the lipoxygenase (LOX) pathway in the tree was also activated by the fungi and produced free fatty acids (Sen et al., 2017). The antioxidant enzymatic activity of *A. sinensis* was enhanced during the early stage of fungal colonization, which triggered a cascade reaction of fatty acid oxidation. This process resulted in a significant increase in the concentrations of JA, salicylic acid (SA), and 1-aminocyclopropyl-1-carboxylic acid (ACC), which induced the expression of sesquiterpene synthase genes and polyketide synthase genes, leading to the continuous accumulation of terpenes and chromones and ultimately forming agarwood (Küpper et al., 2009; Xu et al., 2017; Lv et al., 2019; Zhang P. et al., 2022; Zhang Z. et al., 2022). The infection of *A. sinensis* callus tissue with *Phaeoacremonium rubrigenum* revealed a significant upregulation of the farnesyl diphosphate synthase (FPS) gene, Ses TPS1, and Ses TPS2, which confirmed that the fungi primarily induced the biosynthesis of terpenes through the MVA pathway (Huang et al., 2023). During the biosynthesis of terpenes, significant changes were observed in the levels of protein phosphorylation in 52.9% of the transcription factors (TFs), which phosphorylated the MYB (V-mybavian myeloblastosis viral oncogene homolog), basic leucine zipper (bZIP), and WRKY TFs that were closely related to enzyme activity in the terpene biosynthetic pathway. During the fungal colonization of *A. sinensis*, the accumulation of chromones is promoted by regulating the expression of chalcone synthase (*CHS*). Under the stimulation of *Fusarium solani*, the expression of *CHS* in *A. sinensis* was upregulated after 2 months, and the accumulation of chromones was detected, thus, indicating that this type of component is related to the upregulation of *CHS* expression in *A. sinensis* after fungal stimulation (Chen X. et al., 2017; Chen X. Y. et al., 2017).

In summary, studies have shown that the formation of agarwood is closely related to fungal diversity. Whether the aromatic components are synthesized by fungi, host trees, or both types of organisms has not been thoroughly studied. Future research should focus more on the potential mechanisms of agarwood formation, such as the interaction of functional genes between agarwood, fungi, and the agarwood aromatic components.

## 5 Conclusion

*Aquilaria sinensis* responded strongly during the early stage of induction as shown by the promotion of the formation of aromatic components. The accompanying changes were observed in the structure and function of endophytic fungal communities in the *A. sinensis* xylem. The species richness continuously increased as the time of induction was prolonged. However, the diversity first decreased and then increased. The interactions among fungal species grew closer, and the dominant taxa occupied the key position instead of the rare taxa. A correlation analysis and Mantel’s test identified a correlation between the endophytic fungi and aromatic components. The mixed solution of fungi, inorganic salts and ethylene is an important influence on the formation of agarwood and changes in the fungal communities.

## Data Availability

The datasets presented in this study can be found in online repositories. The names of the repository/repositories and accession number(s) can be found at: https://www.ncbi.nlm.nih.gov/, PRJNA1117010.

## Footnotes

^1^ http://gephi.org/

^2^ https://www.chiplot.online/
