# Supplementary material for: Aromatic components and endophytic fungi during the formation of agarwood in Aquilaria sinensis were induced by exogenous substances
Source: Front Microbiol. 2024 Aug 21;15:1446583. doi: 10.3389/fmicb.2024.1446583 (PMC11371604; doi:10.3389/fmicb.2024.1446583)
Supplement: Supplementary file 1 [file Table_1.DOCX]

**Supplementary materials**

**Table S1** Endophytic fungal diversity indices for different induction treatments and times

| Treatment | Time | Sequences  number (×10^5^) | OTU  number | Shannon  index | Chao1  index | Pielou  index |
| --- | --- | --- | --- | --- | --- | --- |
| T1 | 0M | 1.42 (0.38) | 88 (18) | 3.01 (0.28) | 89 (17) | 0.68 (0.03) |
|  | 3M | 1.40 (0.36) | 153 (15) | 2.53 (0.07) | 202 (12) | 0.50 (0.01) |
|  | 6M | 1.69 (0.04) | 146 (30) | 1.55 (0.56) | 199 (40) | 0.31 (0.10) |
|  | 9M | 2.11 (0.10) | 117 (9) | 2.29 (0.13) | 160 (11) | 0.48 (0.03) |
| T2 | 0M | 0.99 (0.38) | 59 (13) | 2.55 (0.28) | 74 (22) | 0.65 (0.11) |
|  | 3M | 2.16 (0.17) | 110 (3) | 1.62 (0.21) | 183 (10) | 0.34 (0.04) |
|  | 6M | 1.76 (0.13) | 118 (6) | 1.15 (0.13) | 167 (9) | 0.24 (0.03) |
|  | 9M | 1.28 (0.57) | 154 (33) | 2.61 (0.36) | 201 (34) | 0.52 (0.05) |
| T3 | 0M | 0.70 (0.35) | 56 (5) | 2.79 (0.57) | 60 (4) | 0.69 (0.14) |
|  | 3M | 1.59 (0.28) | 171 (14) | 2.83 (0.13) | 221 (25) | 0.55 (0.03) |
|  | 6M | 1.33 (0.39) | 163 (15) | 2.23 (0.28) | 209 (16) | 0.44 (0.05) |
|  | 9M | 1.80 (0.24) | 145 (9) | 2.28 (0.17) | 190 (13) | 0.46 (0.03) |

**Table S2** Two-way ANOVA of endophytic fungal Alpha diversity indices with induction treatments and times

| Index/F-value | Sequence number | OTUs  number | Shannon index | Chao1  index | Palou’s index |
| --- | --- | --- | --- | --- | --- |
| Treatment | 0.813 ns | 2.065 ns | 3.634 * | 0.225 ns | 2.463 ns |
| Time | 2.161 ns | 22.339 ** | 7.957 ** | 31.404** | 14.099 ** |
| Treatment*Time | 1.250 ns | 1.788 ns | 1.857 ns | 0.831 ns | 1.257ns |

**Table S3** A comparison of the alpha diversity indices of endophytic fungal communities in different induction treatments

| Treatment | Sequence number (×10^5^) | OTUs  number | Shannon index | Chao1  index (×10^2^) | Palou’s index |
| --- | --- | --- | --- | --- | --- |
| T1 | 1.64±0.14 a | 126±11 a | 2.35±0.21 ab | 1.61±0.17 a | 0.49±0.05 a |
| T2 | 1.55±0.20 a | 110±13 a | 1.98±0.22 b | 1.57±0.18 a | 0.44±0.06 a |
| T3 | 1.36±0.18 a | 134±14 a | 2.53±0.17 a | 1.70±0.21 a | 0.53±0.04 a |

**Table S4** Topological indices of endophytic fungal co-occurrence networks in different induction treatments and times

| Treatment | | Nodes | Edges | Average degree | Network diameter | Graph density | Modularity | Average clustering coefficient | Average path distance |
| --- | --- | --- | --- | --- | --- | --- | --- | --- | --- |
| T1 | 0M | 71 | 336 | 9.465 | 3 | 0.135 | 0.605 | 0.926 | 1.194 |
|  | 3M | 89 | 445 | 10.000 | 6 | 0.114 | 0.621 | 0.905 | 2.192 |
|  | 6M | 99 | 1129 | 22.808 | 3 | 0.233 | 0.216 | 0.957 | 1.043 |
|  | 9M | 99 | 635 | 12.826 | 4 | 0.131 | 0.618 | 0.947 | 1.376 |
| T2 | 0M | 63 | 286 | 9.079 | 4 | 0.146 | 0.657 | 0.771 | 1.507 |
|  | 3M | 66 | 269 | 8.152 | 3 | 0.125 | 0.702 | 0.956 | 1.199 |
|  | 6M | 77 | 431 | 11.195 | 5 | 0.147 | 0.67 | 0.947 | 1.47 |
|  | 9M | 95 | 1579 | 33.242 | 8 | 0.354 | 0.126 | 0.922 | 1.786 |
| T3 | 0M | 51 | 272 | 10.667 | 4 | 0.213 | 0.391 | 0.917 | 1.343 |
|  | 3M | 62 | 268 | 8.645 | 2 | 0.142 | 0.677 | 0.964 | 1.025 |
|  | 6M | 97 | 742 | 15.299 | 6 | 0.159 | 0.644 | 0.9 | 1.328 |
|  | 9M | 101 | 656 | 12.990 | 6 | 0.13 | 0.695 | 0.886 | 1.619 |
